# Data on public bicycle acceptance among Chinese university populations

**DOI:** 10.1016/j.dib.2019.104946

**Published:** 2019-12-06

**Authors:** Aline Chevalier, Manuel Charlemagne, Leiqing Xu

**Affiliations:** a Tongji University, College of Architecture and Urban Planning, China; b Shanghai Jiao Tong University, UM-SJTU Joint Institute, China

**Keywords:** Bicycle acceptance, Built environment, Shared bikes, Urban cycling, Individual perception, China

## Abstract

This data article provides the data related to the research article entitled “Bicycle acceptance on campus: Influence of the built environment and shared bikes” (Chevalier et al., 2018) [1]. The data is the combination of two distinct datasets: (i) the built-environmental characteristics of the cycling environment and (ii) the answers of 1131 respondents to a structured questionnaire on public bicycle acceptance collected via an on-line survey platform. The survey took place in 2018 and gather respondents spread over five different university campuses in Shanghai (China). The data provides detailed information on individual perception of bicycles in general and Dock-less App-based Shared Bike (DASB) systems in particular. The dataset related to the questionnaire can be split into three types of data; (i) the personal profile and respondent's preferences in terms of transportation; (ii) the perception of cycling and cyclists at a city level and (iii) this perception restricted to the campus area. The association of the cycling environment characteristics and the individual perception of bicycles displayed in each group allow different levels of data analysis to explore the relationship between the built environment and the public acceptance of both the bicycle in general and the DASB in particular.

**Table:** Specifications Table

Subject	Environmental psychology
Specific subject area	Public Bicycle Acceptance
Type of data	Table Chart Figure
How data were acquired	Objective data was obtained by counts and measurements during on-site visits or using campuses maps and an online map application. Subjective data was collected through a questionnaire available via an online survey platform.
Data format	Raw
Parameters for data collection	Objective data relate to measurements of the built environment. Subjective data derive from respondents answers to a questionnaire, mostly using a five-level Likert's scale, to express their perceptions of the bicycle in the city in general and those ones on campus.
Description of data collection	For each campus, data was collected during several on-site visits at different times of the day. Respondents were people who were visiting one of the investigated campuses on a regular basis. After scanning a QR code, each respondent was able to freely answer the questions on his/her personal device.
Data source location	Institution: Tongji University, Shanghai Jiao Tong University, East China Normal University, Donghua University. City/Town/Region: Shanghai Country: China
Data accessibility	With the article
Related research article	Aline Chevalier, Manuel Charlemagne, Leiqing Xu Bicycle acceptance on campus: Influence of the built environment and shared bikes Transportation Research Part D: Transport and Environment https://doi.org/10.1016/j.trd.2019.09.011 Article reference: TRD2133

**Table:** 

**Value of the Data** • The data can be a useful source of information for any campaign, program or transport planning measures that aims to improve public bicycle acceptance, especially in the context of a high bicycle modal share. • This data can be employed for individual statistical analysis and meta-analysis. • The experimental conditions of the data collection can be used as an example of methodology to overcome the complexity of investigations involving multiple contextual and physical factors. • The data was collected in China and has cultural diversity value.

## 1 Data

**Fig. 1:**
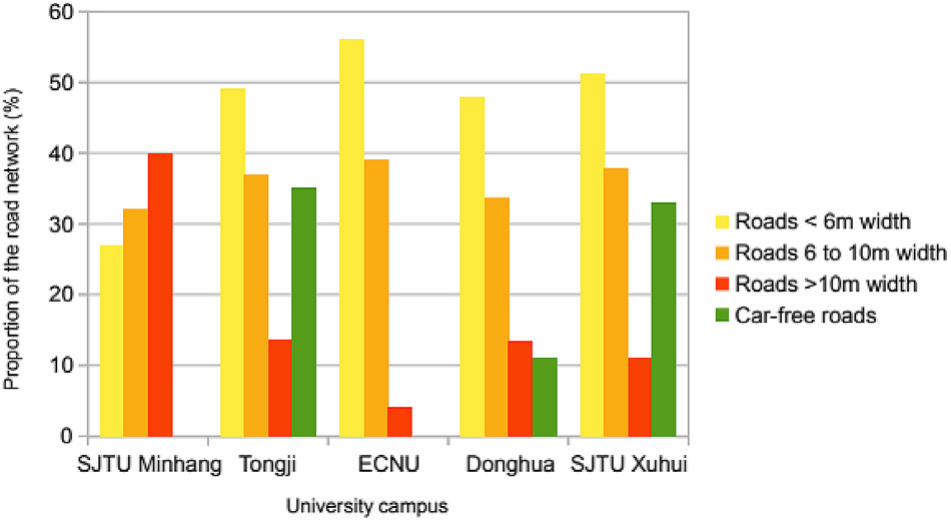
Road network characteristics.

**Fig. 2:**
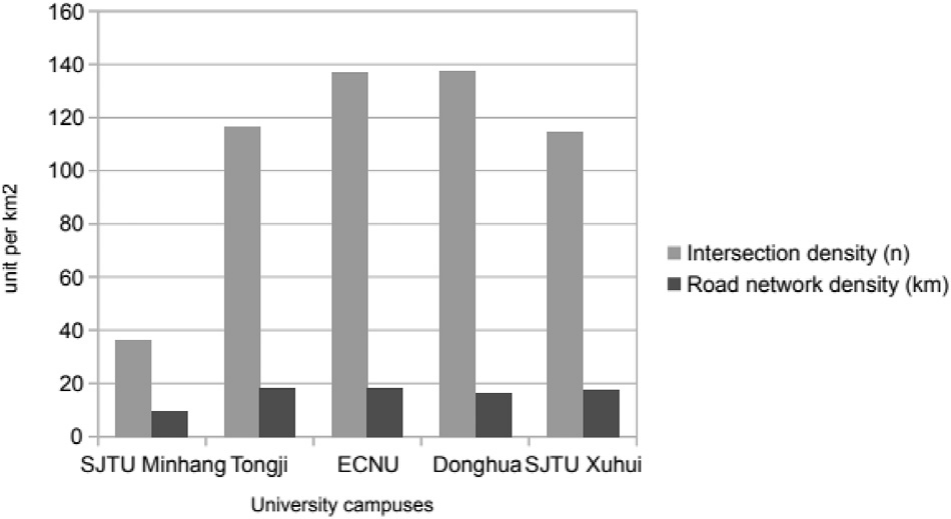
Road network density.

**Fig. 3:**
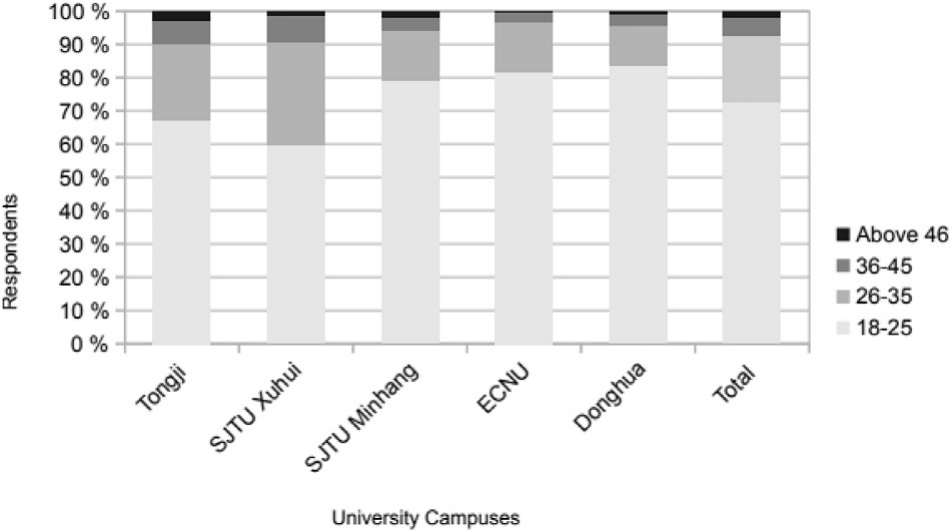
Age distribution of the sample.

**Fig. 4:**
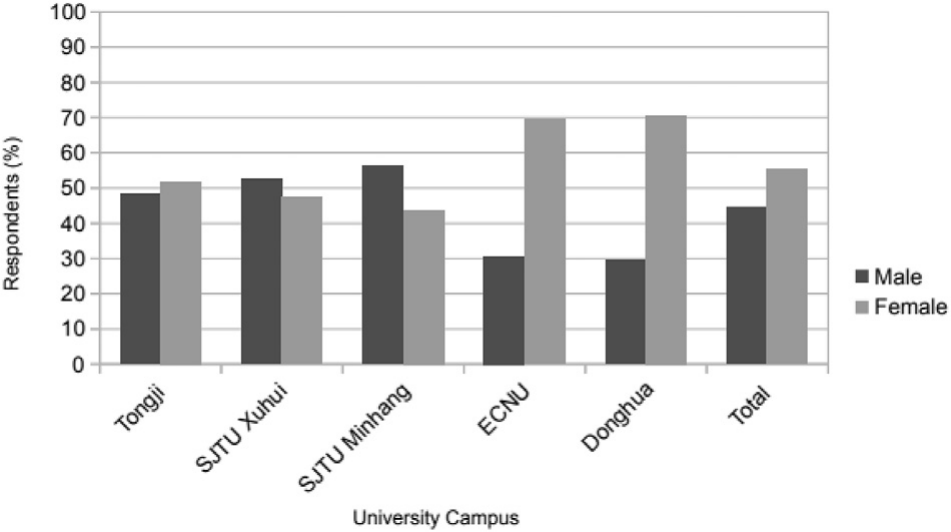
Gender distribution of the sample.

This data article provides the dataset used in a research article entitled “Bicycle acceptance on campus: Influence of the built environment and shared bikes” (Chevalier et al., 2018) [1]. This dataset is composed of two very distinct types of data; objective data, relating to the built environment, and subjective data, reflecting individual perception of urban cycling and the related attitudinal issues. Table 1 exposes the methods that have been used to collect objective data. Table 2 describes the various elements extracted from the structured-questionnaire survey to form the respondents’ profile. Also obtained from the questionnaire, Table 3 presents the questions and related answers that refer to the perception of the bicycle at a city level while Table 4 provides the ones referring to the perception of the bicycle restricted to university campuses. Fig. 1 exhibits the road networks characteristics of the five campuses investigated. Fig. 2 presents the road network density on each of those campuses. Both of these figures display data extracted from the set of objective data. Fig. 3, Fig. 4 present the sample of respondents that provided the answers to the structured-questionnaire survey. Fig. 3 shows the age distribution and Fig. 4 the gender distribution for this sample.

**Table 1:** Built-environmental data collection methods.

Categories	Criteria	Data acquisition
General features	Surface area (km^2^)	Universities' campus maps
	Distance from City-hall (km)	Online map application
	Surrounding land-use	Online map application
Accessibility	Distance from main gate to metro-station (m)	Online map application
	Number of bus-stops within 250 m of campuses	Online map application and on-site counts
	Number of highways	Online map application
Road network	Road density (km/km^2^)	Universities' campus maps and Online map application
	Intersection density (n/km^2^)	Universities' campus maps and Online map application
	3way intersections (%)	Universities' campus maps and Online map application
	4way intersections (%)	Universities' campus maps and Online map application
	Cycle-lanes’ road surface (asphalt/paved/colour)	On-site survey
Road width (Proportion of the total road length on campus)	Less than 6 m (%)	Measured on-site
	From 6 to 10 m (%)	Measured on-site
	Above 10 m (%)	Measured on-site
Campus specificities	Car-free roads (%)	On-site survey
	Shared bikes on parking (% of parked bikes)	On-site counts on bike parking areas at main gates
	Level of greenery	On-site survey and online map application
	Cycle-lanes’ road surface (asphalt/paved/colour)	On-site survey

**Table 2:** Respondent's personal profile.

Categories	Criteria	Form of the answer (unit)
Socio-demographic information	Age	(years old)
	Gender	Male/Female
	Occupation	Student
		Employee
		Self-employed
		Unemployed
		Retired
	Type of occupation	Field or Major when applicable
Travel norms	Usual travel time	One way (min)
	Common transportation	Walking
		Bicycle
		E-bike
		Metro/bus
		Car/taxi
		Other
	Favourite transportation	Walking
		Bicycle
		E-bike
		Metro/bus
		Car/taxi
		Other
Vehicle ownership	Car	Yes/No
	Bicycle	Yes/No
	E-bike	Yes/No
	Motorbike	Yes/No
	Kick-scooter	Yes/No
	Other	Yes/No
Cycling experience	Shared-bikes user	Yes/No
	Riding frequency	Every day
		Every week
		Sometimes
		Never
	Last ride	Within the past few days
		Within the past few weeks
		Within the past few years
		More than ten years ago
		Never rode a bicycle

**Table 3:** Perception of the bicycle at a city level.

Categories	Statement	Form of the answer
City	Bicycles can be an issue for other road users in general	Five-level Likert's scale
	Why can cyclists be an issue for other road users? (When applicable)	They go too fast
		They go too slow
		There is too many of them
		They are unpredictable
		They don't pay enough attention
		They are unaware of danger
		They don't follow traffic rules
		Bikes are often in bad conditions
		Other
	Bicycles belong to motorised or pedestrian traffic	Motorised/Pedestrian
	Shanghai is a bikeable city	Yes/Maybe/No
Shared bikes	The major issue with cyclists on shared bikes is:	They are unskilled cyclists
		They go too slow
		They go too fast
		There is too many of them
	Shared-bike parking is an issue	Five-level Likert's scale
	Why is shared-bike parking an issue? (When applicable)	This is ugly
		It is disturbing pedestrians
		It is disturbing motorists
		It is dangerous
		Other
	Shared-bikes can lead to a waste of bicycles	Five-level Likert's scale
General appreciation for cycling	Bicycles are part of the Chinese culture	Yes/No
	Shared-bikes improved the cycling experience in Shanghai	Yes/No
Individual perception of cycling	It's healthy	Yes/No
	It's fun	Yes/No
	It's fashionable	Yes/No
	It's convenient	Yes/No
	It's tiring	Yes/No
	It's slow	Yes/No
	It's dangerous	Yes/No

**Table 4:** Perception of the bicycle restricted to university campuses.

Categories	Statement	Form of the answer
On campus	Bicycles can be an issue for other road users on campus	Five-level Likert's scale
	Why can cyclists be an issue for other road users? (When applicable)	They go too fast
		They go too slow
		There is too many of them
		They are unpredictable
		They don't pay enough attention
		They are unaware of danger
		They don't follow traffic rules
		Bikes are often in bad conditions
		Other
	Shared-bike parking is an issue	Five-level Likert's scale
Built environment	Road marking can improve the circulation of cyclists	Five-level Likert's scale
	Road surface can improve the circulation of cyclists	Five-level Likert's scale
Social acceptance of the bicycle	Campus should be a privileged location to cycle	Yes/No

## 2 Experimental design, materials, and methods

### 2.1 Objective data

As the built environment was found to play an important role in the cycling experience the aim of our approach was to capture the features of the built environment and include them in the factors relative to public acceptance of bicycles in general and Dock-less App-based Shared Bike (DASB) systems in particular [2]. Although bicycle acceptance has often been studied from a sociological viewpoint, an approach including urban-form factors is much more complex to arrange [3]. In this context, the investigation ground should comply with three major requirements: (i) be within an enclosed area, to ensure a common typology of the urban environment for each group of respondents; (ii) feature a high bicycle modal share allowing the projection into future conditions in the event of an increase in the practice of urban cycling; and (iii) present a high penetration level of DASB. In that regard campuses in Shanghai, a pioneer city for ADBS where the bicycle modal share is among the highest worldwide, represent a perfect investigation ground answering all those three requirements.

The collected information allowed a detailed outline of the urban forms with a special emphasis on the road network as it represents the major element of a cycling environment [4] (Figs. 1 and 2). The environmental characteristics of each campus were evaluated with respect to (i) its surrounding major features of the built environment such as public transport, distance from city centre, residential and commercial areas measured on maps provided by Internet map applications [5]; (ii) the characteristics of their road network with a thorough examination of their morphology (determined from university's campuses maps and on-site investigations); and (iii) the specifics related to internal campus policies evaluated according to the following criteria: proportion of car-free roads, amount of shared-bikes on parking lots, and level of greenery (evaluated from on-site counts) (Table 1).

### 2.2 Subjective data

In addition to the comprehensive dataset of objective data on the built environment, subjective data relating to individual perception of urban cycling were collected through a structured questionnaire accessible via an on-line survey platform. For each campus, this data was collected during several on-site visits at various times of the day. Respondents were selected by an intercept survey method and were previously asked if they were visiting the campus on a regular basis to insure their answers would reflect an accurate appreciation of the investigated environments. After scanning a QR code, each respondent was able to freely answer to the questions, in order to prevent any external influence. Many respondents posted the questionnaire on group-chats or forwarded it through social networks. As a result, our initial database gathered almost 1500 respondents. However when cleaning up the data, respondents were immediately removed if they were related to a campus outside of our investigation. The questionnaire also featured some redundant questions allowing us to withdraw respondents providing inconsistent answers. Although these removals represented close to 25% of the initial database we were left with 1131 respondents.

### 2.3 Structured questionnaire survey

The questionnaire was divided in three parts: (i) definition of the personal profile; (ii) the perception at a city level of bicycles in general and DASB in particular; (iii) that perception of bicycles restricted to the campus area (the Chinese and English version of the questionnaire are available in the Supplementary material).

The personal profile gathers some basic socio-demographic information given by respondents and personal details relating to travel norms (Table 2). The individual perception of bicycles at a city level encompasses various types of information such as an evaluation of the city's bicycle-friendliness or specific attitudinal issues towards two-wheeled vehicles (Table 3). The questions relating to the perception of bicycles on campus provide the respondents' views on the bicycle and its practice based on the surrounding environment (Table 4).

For most of the questions on individual perception, a five-level Likert's scale was used to gather the responses (1-not at all/2-slightly/3-moderately/4-strongly/5-definitely).

### 2.4 Constructing a new variable

As for their evaluation of cycling in terms of personal benefits or draw-backs, people had to pick among a list of seven adjectives (healthy, fun, fashionable, convenient, tiring, slow, dangerous) the one(s) describing their personal appreciation of the practice (Table 3). These personal points of view are gathered under the label ‘Individual perception of cycling*’. Our questionnaire featured simple “yes” or “no” questions relative to personal opinions on the practice. Each person could positively or negatively answer each of those questions. Since they were all independent of each others and a respondent could feel for instance that cycling is both ‘dangerous’ and ‘healthy’ we decided to encode all of them into a single variable IndividualPerception*. To construct this new variable we used a simple bijective map defined as follows:

$$f\text{(x)}=\sum_{i=\text{0}}^{6}x_{i}\times 2^{i}\text{,}$$

where x = (x 0, x 1, x 2, x 3, x 4, x 5, x 6) and x i, 0 ≤ i ≤ 6 represents the respondent's answer, defined as 0 or 1, to the bicycle practice being ‘dangerous’, ‘tiring’, ‘slow’, ‘convenient’, ‘fashionable’, ‘fun’, and ‘healthy’, respectively. By definition our function f is strictly increasing and maps any combination of answers to a different positive integer. In particular prominently negative feelings map to small integers while positive ones map to larger numbers. As a result this simple encoding allows us to better interpret the personal evaluation of the respondents towards the personal benefits and draw-backs of cycling. This new variable was then added into the dataset.

### 2.5 Description of the sample

Due to the specific experimental conditions, the vast majority of the respondents are students. While this category represents roughly 80% of the sample, university staff constitutes 10%, and outsiders visiting the campus on a regular basis another 10%. This is clearly reflected by the distribution across age groups: about 70% of the respondents are between 18 and 25 years old, 20% between 26 and 35, and only 10% above 36 (Fig. 3).

As for gender, it is relatively balanced over the entire sample with 45% males and 55% females but varies greatly according to the university. While SJTU is mainly represented by males, respondents from the ECNU and Donghua are mostly females (Fig. 4). This gender distribution follows the official figures displayed by the various universities with a greater male population following a scientific curriculum and a majority of female students in humanities, literary or artistic fields. On a more global scale, the slightly higher proportion of female in the sample reflects the general tendency observed in China and worldwide [6]. Furthermore, in our sample, the vast majority of our respondents use bicycles very regularly with 55% cycling at least once a week. Only less than 10% of them never ride a bicycle while more than 85% are DASB users and 50% are bicycle owners.]
