# Supplementary material for: Data on public bicycle acceptance among Chinese university populations
Source: Data Brief. 2019 Dec 6;28:104946. doi: 10.1016/j.dib.2019.104946 (PMC6921136; doi:10.1016/j.dib.2019.104946)
Supplement: Multimedia component 2 [file mmc2.pdf]

Public Bicycle Acceptance

1/ What is your gender? Male □ Female □

2/ What is your nationality? ______________________

3/ How old are you? <18 □ 18-25 □ 26-35 □ 36-45 □ 46-55 □ >56 □

4/ Which university campus do you visit most often?

TongJi (Siping) □ JiaoTong (Xuhui) □ JiaoTong (Minhang) □ ECNU □ Donghua □

Other □____

5/ What is your occupation?

Student □ University staff □ Work out □ Retired □ Unemployed □

6/ How much time do you travel from home to work/school? ________h________min

7/ What is your <u>FAVORITE</u> way of travelling?

Walking □ Bicycle□ Electric bike □ Bus □ Metro □ Taxi □ Car □

Other □ ____

Why?

It is cheaper □ I have more freedom □ This is more comfortable □ It is quicker □

It is less dangerous □ It is more enjoyable/exciting □ It is more convenient □

8/ What is your <u>most common/usual</u> way of travelling ?

Walking □ Bicycle□ Electric bike □ Bus □ Metro □ Taxi □ Car □

Other □ ____

Why?

It is cheaper □ I have more freedom □ It is more comfortable □ It is quicker □

It is less dangerous □ It is more enjoyable □ It is more convenient □

9/ Do you think Shanghai is a bikable city? Yes □ Maybe □ No □

10/ Do you own a car? Yes □ No □

11/ Do you own a two-wheeler vehicle? Yes □ No □ **Please specify:**

Bicycle □ Electric bike □ Motorbike □ Kick scooter □ Mono-wheel □

Other □ ____

12/ How often do you ride a two-wheeler vehicle ?

Every day □ Every week □ Sometimes □ Never □

13/ When did you last ride a bicycle ?

Within few days □ Within few weeks □ Few years ago □ More than 10 years ago □

14/ If you never ride any bicycle or E-bike, what is the main reason?

It's dangerous □ I don't know how to ride □ It is too tiring □ It is too polluted □

The weather is often not good enough □ I don't want my bike to get stolen □

Other □ ____

15/ Do you think cyclists on pedal bicycles can be an issue for other road users in the city?

Not at all □ Slightly □ Moderately □ Strongly □ Very strongly □

If yes, why?

They go too fast □ They go too slow □ There is too many of them □

They are unpredictable □ They do not pay enough attention □ They are unaware of danger □

Bikes are often in bad conditions □ They do not respect traffic rules □

Other □____

16/ Do you think bicycles belong to : *motorized* □ **or** *pedestrian* □ **traffic?**

17/ In the following elements, which one would make you feel more at ease with two-wheelers vehicles?

More bicycle lanes □ Better bicycle lanes □ More separation in traffic □

Other □____

18/ In general, with what kind of road users do you feel the most uncomfortable?

Cars □ Bicycles □ Electric bikes □ Buses □ Taxis □ Pedestrians □

Why?

They go too fast □ They go too slow □ There is too many of them □

They are unpredictable □ They do not pay enough attention □ They are unaware of danger □

Bikes are often in bad conditions □ They do not respect traffic rules □

Other □ ____

19/ Do you think cyclists on pedal bicycles are an issue for other road users on campus?

Not at all □ Slightly □ Moderately □ Strongly □ Very strongly □

Why?

They go too fast □ They go too slow □ There is too many of them □

They are unpredictable □ They do not pay enough attention □ They are unaware of danger □

Bikes are often in bad conditions □ They do not respect traffic rules □

Other □____

20/ Do you think road marking can improve the circulation of cyclists on campus?

Not at all □ Slightly □ Moderately □ Strongly □ Very strongly □

21/ Do you think road surface can improve the circulation of cyclists on campus?

Not at all □ Slightly □ Moderately □ Strongly □ Very strongly □

22/ Do you use bicycle sharing systems (such as Mobike or Ofo)? Yes ▢ No ▢

23/ What do you think is the major issue with cyclists on shared bicycles?

Cyclists on shared bikes are unskilled ▢ They go too slow ▢ They go too fast ▢

There is too many cyclists ▢ Other ▢ ______________________________

24/ Do you think shared-bikes systems can lead to a waste of bicycles?

Not at all ▢ Slightly ▢ Moderately ▢ Strongly ▢ Very strongly ▢

25/ Are you bothered by bicycle parking?

Not at all ▢ Slightly ▢ Moderately ▢ Strongly ▢ Very strongly ▢

If yes, why?

This is ugly ▢ It is disturbing pedestrians ▢ It is disturbing motorists ▢

It's dangerous ▢ Other ▢ ______________________________

26/ What is your opinion on cycling?

It's healthy: Yes ▢ No ▢

It's fun: Yes ▢ No ▢

It's fashionable: Yes ▢ No ▢

It's convenient: Yes ▢ No ▢

It's tiring: Yes ▢ No ▢

It's slow: Yes ▢ No ▢

It's dangerous: Yes ▢ No ▢

Bicycles are part of the Chinese culture: Yes ▢ No ▢

Campus should be a privileged location to cycle: Yes ▢ No ▢

Shared-bikes improved the cycling experience in Shanghai: Yes ▢ No ▢

Thank you very much for your collaboration!
