# Supplementary material for: Data on public bicycle acceptance among Chinese university populations
Source: Data Brief. 2019 Dec 6;28:104946. doi: 10.1016/j.dib.2019.104946 (PMC6921136; doi:10.1016/j.dib.2019.104946)
Supplement: Multimedia component 3 [file mmc3.pdf]

# 如何看待自行车

**1/ 性别:** 男 □ 女 □

**2/ 您的国籍？** 中国人 □ 外国人 □

**3/ 您的年龄：** <18 □ 18-25 □ 26-35 □ 36-45 □ 46-55 □ >56 □

**4/ 您的大学是什么?** 同济大学 □ 交通大学(闵行校区) □ 交通大学(徐汇校区) □

其他 □ ____________________________________________

**5/ 您的职业是什么?** 学生 □ 教师及学校工作人员 □ 非校内人员 □ 退休 □

**6/ 您从家到单位/学校花多长时间**__________小时__________分钟

**7/ 您在城市里<u>最喜欢</u>的交通方式是什么? (单选)**

步行□ 自行车□ 电动车 □ 公交车 □ 地铁□ 出租□ 自驾车□
其他 □ ____________________________________________

**为什么? (单选)**

经济实惠□ 更加自由 □ 更加舒适□ 更加快捷 □

更加安全□ 更加享受/刺激 □ 更加方便 □ 可控 □

**8/ 您最常用的城市交通方式是什么? (单选)**

步行□ 自行车/电动车 □ 公交车 □ 地铁□ 出租□ 自驾车□
其他 □ ____________________________________________

**为什么? (单选)**

经济实惠□ 更加自由 □ 更加舒适□ 更加快捷 □

更加安全□ 更加享受/刺激 □ 更加方便 □ 可控 □

**9/ 您认为上海是一个适合骑自行车的城市吗？** 是 □ 也许/我不知道 □ 不是 □

**10/ 您有私家车吗？** 是的 ☐ 没有 ☐

**11/ 您有单车吗?** 是的 ☐ 没有 ☐

**您的单车是:**
自行车□ 电动自行车 □ 电动摩托 □ 滑板车 □
三轮车□ 电动三轮车□ 电动滑板□ 单轮车 □
其他 □ ____________________________________________

**12/ 您使用单车的频率?**

每天□ 每周 □ 偶尔 □ 从不 □

**13/ 您上次骑车是在什么时候?**

在过去的几天里 □ 几周前□ 几月前□ 几年前□ 10 多年前 □ 从不 □

**14/ 如果您从未骑过车，最主要原因是什么? (单选)**

危险□ 不会骑车□ 太累 □ 空气太脏 □ 天气经常不好□ 我不想单车被偷□

其他 □ ________________________________________________________________

**15/ 您认为骑车的人对其他开车的人或行人造成影响吗？**

非常严重影响 □ 严重影响 □ 一般影响 □ 略微影响 □ 不影响 □

**如果是，为什么? (单选)**

他们不够小心 □ 他们速度太快 □ 他们不遵守交通规则 □ 他们意识不到危险 □

其他 □________________________________________________________________

**16/ 如果没有自行车道，您认为自行车属于: 机动车** □ 还是 **步行者** □ **?**

**17/ 以下哪些情况会让你更喜欢骑车？(单选)**

更多的自行车道 □ 更好的自行车道 □ 马路更多划分道路 □

其他□________________________________________________________________

**18/ 您认为哪种交通工具的使用者最让你心烦？(单选)**

小车□ 自行车 □ 助动车□ 公交车□ 出租车□ 行人□

**为什么?**

他们速度太快 □ 他们不可预测□ 他们不遵守规则□

其他□ ________________________________________________________________

**19/ 您认为校园骑车的人对您构成干扰吗?**

非常严重干扰□ 严重干扰□ 一般干扰 □ 略微干扰□ 不干扰□

**为什么? (单选)**

速度太快 □ 不可预测 □ 不遵守规则□ 骑车的人太多□

其他 □________________________________________________________________

**20/ 您认为校园对道路重新标线有助于提高校园交通吗？**

极大帮助□ 很大帮助 □ 一般帮助□ 略微帮助□ 没有帮助 □

**21/ 你对校园路面认为适合骑自行车吗？**

极大合适□ 很大合适 □ 一般合适□ 略微合适□ 不合适 □

**22/ 您使用共享单车吗？** 用□ 不用□

**23/ 您认为共享单车使用者最大的问题是什么？(单选)**

技术太差□ 速度太慢□ 速度太快□ 骑车的人太多□

其他 □ ________________________________________________________________

**24/ 您认为共享单车是对自行车的浪费吗？**

非常严重的浪费□ 严重的浪费□ 一般浪费□ 略微有点浪费□ 不是浪费□

**25/ 您讨厌他人的自行车停放吗？**

极其 □ 非常 □ 一般□ 稍微有点□ 一点也不 □

**如果讨厌，为什么？(单选)**

不好看□ 阻碍步行□ 阻碍开车的人□ 危险□

其他 □ ________________________________________________________________

**26/ 您如何看待骑车？**

**有益健康:** 是 □ 否□

**有趣:** 是 □ 否□

**时髦:** 是 □ 否□

**方便:** 是 □ 否□

**很累:** 是 □ 否□

**缓慢:** 是 □ 否□

**危险:** 是 □ 否□

**骑车是中国文化的一部分：** 是□ 否□

**校园应该鼓励骑车：** 是□ 否□

**在上海，您觉得使用共享单车更能享受到骑行乐趣吗？** 是□ 否□

非常感谢您的合作！
